# Identification of Novel Natural Products as Effective and Broad-Spectrum Anti-Zika Virus Inhibitors

**DOI:** 10.3390/v11111019

**Published:** 2019-11-02

**Authors:** Yaning Gao, Wanbo Tai, Ning Wang, Xiang Li, Shibo Jiang, Asim K. Debnath, Lanying Du, Shizhong Chen

**Affiliations:** 1 Department of Natural Medicines, School of Pharmaceutical Sciences, Peking University, Beijing 100191, China; gyn9225@bjmu.edu.cn (Y.G.); lixiang2013@bjmu.edu.cn (X.L.); 2 Lindsley F. Kimball Research Institute, New York Blood Center, New York, NY 10065, USA; wtai@nybc.org (W.T.); NWang@nybc.org (N.W.); sjiang@nybc.org (S.J.); 3 Key Laboratory of Medical Molecular Virology (MOE/NHC/CAMS), School of Basic Medical Sciences, Shanghai Medical College, Fudan University, Shanghai 200032, China

**Keywords:** flaviviruses, Zika virus, natural products, antiviral inhibitors, broad-spectrum activity, combinatorial effect

## Abstract

Zika virus (ZIKV) infection during pregnancy leads to severe congenital Zika syndrome, which includes microcephaly and other neurological malformations. No therapeutic agents have, so far, been approved for the treatment of ZIKV infection in humans; as such, there is a need for a continuous effort to develop effective and safe antiviral drugs to treat ZIKV-caused diseases. After screening a natural product library, we have herein identified four natural products with anti-ZIKV activity in Vero E6 cells, including gossypol, curcumin, digitonin, and conessine. Except for curcumin, the other three natural products have not been reported before to have anti-ZIKV activity. Among them, gossypol exhibited the strongest inhibitory activity against almost all 10 ZIKV strains tested, including six recent epidemic human strains. The mechanistic study indicated that gossypol could neutralize ZIKV infection by targeting the envelope protein domain III (EDIII) of ZIKV. In contrast, the other natural products inhibited ZIKV infection by targeting the host cell or cell-associated entry and replication stages of ZIKV. A combination of gossypol with any of the three natural products identified in this study, as well as with bortezomib, a previously reported anti-ZIKV compound, exhibited significant combinatorial inhibitory effects against three ZIKV human strains tested. Importantly, gossypol also demonstrated marked potency against all four serotypes of dengue virus (DENV) human strains in vitro. Taken together, this study indicates the potential for further development of these natural products, particularly gossypol, as the lead compound or broad-spectrum inhibitors against ZIKV and other flaviviruses, such as DENV.

## 1. Introduction

Zika virus (ZIKV) is a mosquito-borne flavivirus in the same genus as other important human pathogens, including dengue virus (DENV), West Nile virus (WNV), yellow fever virus (YFV), Japanese encephalitis virus (JEV), and tick-borne encephalitis virus (TBEV) [1]. ZIKV was originally isolated in a rhesus macaque in 1947 [2], but this virus has only recently claimed worldwide attention owing to its close association with congenital Zika syndrome (CZS), as represented by microcephaly, fetal demise, central nervous system abnormalities, and other neurological complications [3,4,5,6,7]. No antiviral therapeutics for the treatment of ZIKV-associated human diseases, particularly congenital syndrome and fetal death, have been approved. This calls for the development of safe and effective therapeutic agents against ZIKV infection in humans.

The genome of ZIKV encodes a polyprotein, which is then cleaved by cellular and viral proteases to form three structural proteins, including capsid (C), precursor of membrane/membrane (prM/M), and envelope (E), as well as seven nonstructural proteins, including NS1, NS2A, NS2B, NS3, NS4A, NS4B, and NS5 [8]. The life cycle of ZIKV involves several crucial steps, including viral attachment to target cell receptors or cofactors, receptor-mediated endocytosis (viral entry), virus–endosomal membrane fusion, and postentry or post-translation stages [9,10]. In this life cycle, E protein plays a key role in viral entry into target cells and subsequent fusion of virus and cell membranes; thus, ZIKV E protein serves as an important therapeutic target against ZIKV infection [11,12].

In addition to ZIKV, other flaviviruses, such as DENV, also cause significant problems for humans. Four antigenic serotypes of DENV (DENV-1–4) lead to dengue disease (dengue fever, dengue hemorrhagic fever, or dengue shock syndrome), with cases increasing annually [13,14]. Therefore, the development of broad-spectrum antiviral inhibitors will be useful for the treatment of infections caused by ZIKV and other flaviviruses, including DENV.

In this study, we have identified four anti-ZIKV inhibitors after screening a natural product library, three of which (gossypol, digitonin, and conessine) have not been reported previously against ZIKV infection. We have demonstrated the broad-spectrum activity of these natural products, particularly gossypol, against multiple ZIKV strains and all four DENV serotypes. We have further identified the mechanisms of action and potential targets of these natural products and revealed the enhanced combinatorial effects of gossypol with other natural products in inhibiting ZIKV infection. Overall, our study opens the door for further exploration and development of the identified natural products as the lead compound or broad-spectrum anti-ZIKV and anti-flavivirus inhibitors.

## 2. Materials and Methods

### 2.1. Cells and Viruses

**Figure 1:**
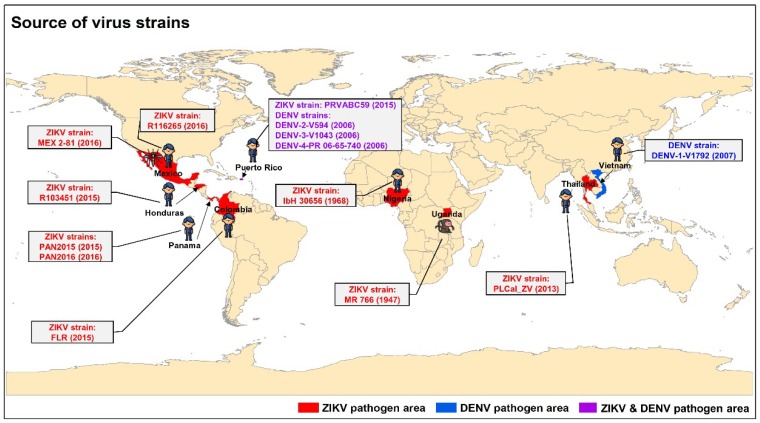
Schematic map for the source of Zike virus (ZIKV) and dengue virus (DENV)-1–4 strains used in the studies. ZIKV only, DENV only, and ZIKV–DENV coinfections are shown in red, blue, and purple, respectively. Countries with isolated ZIKV and DENV strains from different hosts (human, mosquitos, and monkeys) are illustrated. The letters in parentheses indicate the year of ZIKV or DENV isolation.

Vero E6 and LLC-MK2 cells (ATCC, Manassas, VA, USA) were maintained in Dulbecco’s modified Eagle’s medium (DMEM) supplemented with 8% fetal bovine serum (FBS) and penicillin and streptomycin (P/S). C6/36 cells (ATCC) were maintained in Eagle’s minimal essential medium (EMEM) supplemented with 5% FBS and P/S. ZIKV strains, including human strains PAN2016 (2016/Panama), R116265 (2016/Mexico), PAN2015 (2015/Panama), FLR (2015/Colombia), R103451 (2015/Honduras), PRVABC59 (2015/Puerto Rico), PLCal_ZV (2013/Thailand), and IbH 30656 (1968/Nigeria), mosquito strain MEX 2–81 (2016/Mexico), and rhesus macaque strain MR 766 (1947/Uganda) (Figure 1), were used in the studies. These ZIKV strains were cultured in Vero E6 cells, and viral titers were detected by a standard plaque-forming assay [15]. DENV human strains, including type 1: DENV-1-V1792 (2007/Vietnam), type 2: DENV-2-V594 (2006/Puerto Rico), type 3: DENV-3-V1043 (2006/Puerto Rico), and type 4: DENV-4-PR 06-65-740 (2006/Puerto Rico) (Figure 1), were cultured in C6/36 cells, and the viral titers were determined by plaque-forming assay.

### 2.2. Antiviral Activity of Natural Products

A plaque reduction inhibition assay was carried out to measure the inhibitory activity of natural products in a natural product collection library from MicroSource Discovery Systems (Gaylordsville, CT, USA) against infections of ZIKV and DENV, as previously described [15,16,17,18]. Briefly, ZIKV (strain PAN2016, 70~100 plaque-forming unit (PFU); multiplicity of infection (MOI) ~0.001) was incubated with 2-fold serial dilutions of natural products (including curcumin, digitonin and conessine, as well as anti-ZIKV compound control, bortezomib) or DMSO (0.4% vol/vol) control at 37 °C for 1 h. The compound–virus mixtures were then transferred to Vero E6 cells (10^5^/well) and incubated at 37 °C for 1 h. For gossypol, ZIKV (strain PAN2016, ~2.5 × 10^3^ PFU; MOI ~0.025) was incubated with serial dilutions of this natural product at 37 °C for 1 h, and the unbound gossypol was removed by centrifugation after addition of 3% PEG-6000. Gossypol-treated ZIKV was then incubated with Vero E6 cells at 37 °C for 1 h. The cells were washed thoroughly with PBS and overlaid with DMEM containing 1% carboxymethyl cellulose and 2% FBS, followed by in vitro culture at 37 °C for 4–5 days, and then stained with 0.5% crystal violet. The inhibitory activity of all natural products tested against DENV-1–4, as described above, was performed following the above procedures, except that LLC-MK2 cells were used for infection, and cells were cultured at 37 °C for 14–16 days before staining with 0.5% crystal violet. The 50% inhibitory concentration (IC_50_) and IC_90_ of natural products were calculated based on the concentrations at 50% and 90% plaque reduction, respectively, using the CalcuSyn computer program, as described before [17].

### 2.3. Detection of In Vitro Cytotoxicity

The cytotoxicity of natural products in Vero E6 for ZIKV or LLC-MK2 cells for DENV-1–4 was evaluated using the Cell Counting Kit-8 (CCK8, Sigma, St. Louis, MO, USA), according to the manufacturer’s instructions. Briefly, 2-fold serial dilutions of each of the natural products (100 µL/well) were added to equal volumes of cells (2.0 × 10^4^/well) in 96-well plates and cultured at 37 °C for 3 days. The cells were then incubated with CCK8 solution and absorbance was measured at 450 nm (A450 value) using a microplate reader (Infinite F200PRO, Tecan, Morrisville, NC, USA). The 50% cytotoxic concentration (CC_50_) of natural products was calculated based on the percent cytotoxicity using the CalcuSyn computer program [19,20]. The combinatorial cytotoxicity of gossypol with other natural products to Vero E6 cells was detected using a similar approach as described above, except for mixing gossypol with one of the natural products (curcumin, digitonin, conessine, or bortezomib) throughly before adding them to the cells.

### 2.4. Time-of-Addition Experiment

This experiment was carried out as previously described, with some modifications [21,22,23,24,25,26,27]. Briefly, Vero E6 cells (10^5^/well) or ZIKV were incubated at different infection steps as described below, with or without the tested natural products at the specified concentrations of 15 µM for gossypol, 30 µM for curcumin, 7.5 µM for digitonin, or 30 µM for conessine for 1 h before ZIKV infection, 1 h after infection, or the same time during infection. Anti-ZIKV compounds, such as temoporfin [28], 25-hydroxycholesterol [29], bortezomib [16], and NITD008 [30], were included as controls for these steps. After the culture of the ZIKV- or compound-treated cells at 37 °C for 4–5 days, plaques were visualized with crystal violet staining, as described above, and the percent inhibition of natural products was calculated. Specifically, the following six stages of ZIKV infection were tested: (a) pretreatment of ZIKV (PAN2016, ~2.5 × 10^3^ PFU; MOI ~0.025) with natural products at 37 °C for 1 h before incubation with cells; (b) pretreatment of cells with natural products at 37 °C for 1 h before incubation with ZIKV (PAN2016, ~100 PFU; MOI ~0.001); (c) cotreatment of cells with ZIKV (PAN2016, ~300 PFU; MOI ~0.003) and natural products at 4 °C for 1 h; (d) cotreatment of cells, ZIKV (PAN2016, ~100 PFU; MOI ~0.001), and natural products at 37 °C for 1 h; (e) preincubation of cells with ZIKV (PAN2016, ~300 PFU; MOI ~0.003) at 4 °C for 1 h and then incubation with natural products at 37 °C for 1 h; and (f) preincubation of ZIKV (PAN2016, ~100 PFU; MOI ~0.001) and cells at 37 °C for 1 h, followed by incubation with natural products at 37 °C for 1 h.

### 2.5. ELISA

The binding between natural products and ZIKV full-length E protein (Aviva Systems Biology, San Diego, CA, USA) or envelope protein domain III (EDIII) protein (E residues 298–409 fused with a C-terminal human Fc) [15] was carried out by ELISA, as previously described [15,17,31,32]. Briefly, ELISA plates were precoated with the proteins described above at 4 °C overnight and blocked with 2% fat—free milk at 37 °C for 2 h. Serial dilutions of natural products or DMSO (negative control) were then added to the plates and incubated at 37 °C for 2 h. The plates were washed with PBS containing Tween-20 (PBST) and incubated at 37 °C for 2 h with ZIKV EDIII-specific human monoclonal antibody (mAb) ZKA64-LALA (0.5 µg/mL) for binding to ZIKV full-length E and EDIII proteins. The plates were washed with PBST and incubated with horseradish peroxidase (HRP)-conjugated anti-human IgG-Fab (1:3000, Abcam, Cambridge, MS, USA) antibody at 37 °C for 1 h. The 3,3′,5,5′-tetramethylbenzidine (TMB) substrate (Sigma) was added to the plates, and the reaction was stopped by 1 N H_2_SO_4_. Absorbance at 450 nm (A450 value) was measured by ELISA microplate reader (Tecan). EC_50_ (50% effective concentration) was calculated based on the CalcuSyn computer program, as described above [20].

To determine the ability of gossypol to inhibit binding between ZIKV EDIII protein and EDIII-specific human mAbs (SMZAb5, ZKA64-LALA, ZV-67, or Z004) or EDI/II-specific human mAb control (ZKA78) [33], ELISA was carried out, as described above, except that serially diluted gossypol or DMSO (negative control) was added in the presence of mAbs (0.5 µg/mL), followed by sequential incubation with HRP-conjugated anti-human IgG-Fab antibody and TMB substrate and detection for A450 value. The percent inhibition of natural products was calculated, and IC_50_ (concentration causing 50% reduction in EDIII-mAb binding) was obtained using the CalcuSyn computer program [20], as described above.

### 2.6. Surface Plasmon Resonance (SPR)

The interactions between natural products and ZIKV full-length E were analyzed at 25 °C using the Biacore T200 system (GE Healthcare, Port Washington, NY, USA), as previously described [32,34]. Briefly, ZIKV E was immobilized on a sensor chip (CM5) using the Amine Coupling Kit (GE Healthcare). Natural products at various concentrations were injected as analytes, and PBS-P (20 mM phosphate buffer containing 2.7 mM KCl, 137 mM NaCl, and 0.05% surfactant P20, pH 7.4) was used as the running buffer. The data were analyzed using Biacore evaluation software (T200 version 1.0), and the curve was fitted with a 1:1 binding model.

### 2.7. Combinatorial Effects of Gossypol with Other Natural Products against ZIKV Infection

The potential combinatorial effect of gossypol with other natural products was carried out as previously described [25,26]. Briefly, ZIKV (strain PAN2016, FLR, or PRVABC59, 2.5 × 10^3^ PFU; MOI ~0.025) was incubated with serially diluted gossypol at 37 °C for 1 h, and the unbound gossypol was removed by centrifugation after addition of 3% PEG-6000. Gossypol-treated ZIKV was incubated with Vero E6 cells at 37 °C for 1 h in the presence of DMEM containing serial dilutions of each of the other three natural products identified, such as curcumin, digitonin, and conessine, or anti-ZIKV compound control (bortezomib). The unbound viruses and natural products were removed, the cells were cultured at 37 °C for 4–5 days, and stained with 0.5% crystal violet. The natural products without combinations were used as controls. The IC_50_ values of natural products were calculated using the CalcuSyn computer program [20], as described above.

The natural products were then analyzed for combinatorial effects based on the combination index (CI) [20] and IC_50_ values using the CalcuSyn computer program, as previously described [25,35,36]. Specifically, CI values < 1 and > 1 indicate synergy and antagonism, respectively. Synergy was further identified as five different categories: CI values <0.1, 0.1–0.3, 0.3–0.7, 0.7–0.85, and 0.85–0.90 indicate very strong synergism, strong synergism, synergism, moderate synergism, and slight synergism, respectively. Fold enhancement of anti-ZIKV potency is expressed as the ratio of molar concentrations of natural products tested alone and in the mixture using the formula ((IC_50_ alone)/(IC_50_ in the mixture)—1).

## 3. Results

### 3.1. Identification of Lead Natural Products with Broad-Spectrum Activity against Multiple ZIKV Strains

**Figure 2:**
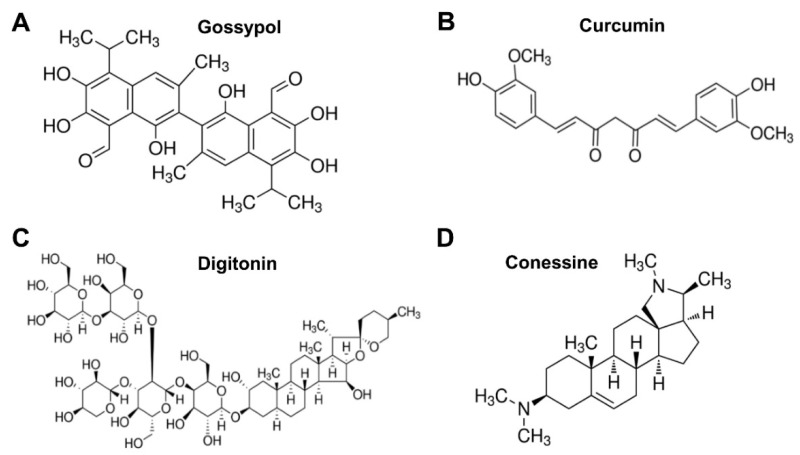
Structure of four lead natural products. Gossypol (**A**), curcumin (**B**), digitonin (**C**), and conessine (**D**) were initially identified as anti-ZIKV (strain PAN2016) inhibitors.

Using a plaque-based assay, we initially screened 720 natural products (at 20 µM of concentration) from a natural product library for their inhibitory activity against infection of a recent ZIKV human strain (PAN2016). Based on Table 1, four “hit” natural products, including gossypol, curcumin, digitonin, and conessine (Figure 2A–D), were selected, since they demonstrated inhibitory activity against ZIKV infection with no obvious cytotoxicity in Vero E6 cells when observed under a microscope. These natural products were subsequently ordered from Sigma with ≥ 95% purity, and tested for their cytotoxicity using a cell-based cytotoxicity assay (CCK8 kit), with CC_50_ values ranging from 14.17 to 323.71 µM (Table 2, Figure S1). Among these natural products, curcumin has been previously reported to inhibit ZIKV infection [37], whereas the other three natural products have not been previously reported to have anti-ZIKV activity. Gossypol, curcumin, digitonin, and conessine were retested to confirm their anti-ZIKV (strain PAN2016) activity, with IC_50_ values of 3.48, 13.67, 4.31, and 9.75 µM, respectively (Table 2). The IC_90_ values of these natural products against ZIKV PAN2016 strain were also calculated, equal to about 6.91, 47.69, 7.91, and 31.82 µM, respectively, for gossypol, curcumin, digitonin, and conessine (Figure S1), which were slightly higher than the respective IC_50_ values, but much lower than the corresponding CC_50_ values.

The identified natural products were further studied for their broad-spectrum activity against nine additional ZIKV strains, including those isolated from different hosts, namely humans, mosquitos, and rhesus macaques, at different time periods (1947–2016) in different countries, including Mexico, Panama, Columbia, Honduras, Puerto Rico, Thailand, Nigeria, and Uganda (Figure 1). The results showed that these natural products could inhibit infections of all nine ZIKV strains tested with various IC_50_ values (Table 2). Gossypol exhibited the most potent inhibitory activity for IC_50_ values, ranging from 0.21 to 4.31 µM, against all nine ZIKV strains tested (Table 2). It was also more potent than bortezomib, the anti-ZIKV previously reported compound as an active compound control, against all ZIKV strains tested (Table 2). The IC_90_ values of these natural products against the selected ZIKV strain, PRVABC59, were calculated, among which gossypol had the lowest IC_90_ value (about 8.81 µM, slightly higher than its IC_50_ value but lower than its CC_50_ value) (Figure S2), confirming its strong and broad-spectrum anti-ZIKV activity.

Comparison of ZIKV E protein sequences revealed that most of the amino acid sequences were highly conserved, but that some variations occurred among the 10 ZIKV strains used for evaluation of the inhibitory activity of natural products, including the PAN2016 strain tested earlier (Figure S3). The above data demonstrate that the identified natural products, particularly gossypol, were able to block infections of divergent human, mosquito, and monkey ZIKV strains isolated from different time periods and countries, including six recent ZIKV human strains, confirming their broad-spectrum anti-ZIKV activity.

### 3.2. Inhibition Mechanisms of Lead Natural Products against ZIKV Infection

**Figure 3:**
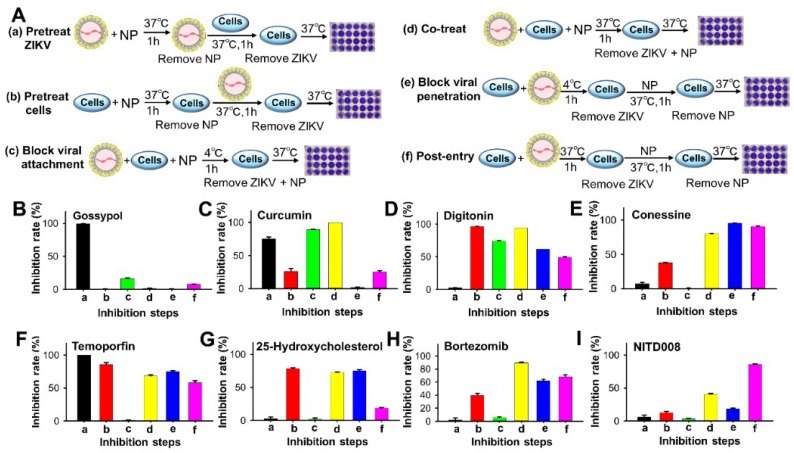
Time-of-addition experiment to test the ability of natural products to block ZIKV infection at different steps of the viral life cycle [21]. (**A**) The time-of-addition experiment was performed in Vero E6 cells, and specific procedures are illustrated in detail in (**a**–**f**). (**a**) Pretreatment of ZIKV: ZIKV was incubated with one of the natural products (NPs; including gossypol, curcumin, digitonin, and conessine) at 37 °C for 1 h. After removal of the unbound NPs, the NP-treated ZIKV was incubated with cells at 37 °C for 1 h, followed by culture of the cells at 37 °C for 4–5 days before calculation of plaques. (**b**) Pretreatment of cells: cells were preincubated with one of the NPs at 37 °C for 1 h, and the unbound NPs were then removed, followed by addition of ZIKV and incubation of cells at 37 °C for 1 h. After removal of the unbound ZIKV, the cells were cultured and plaques were calculated as in (**a**). (**c**) Blockage of ZIKV attachment: cells were incubated with ZIKV at 4 °C for 1 h to allow ZIKV attachment, but not fusion between ZIKV and cell membranes, in the presence of one of the NPs. After removal of the unbound ZIKV and NPs, the cells were cultured and plaques were calculated as in (**a**). (**d**) Cotreatment of ZIKV and cells: cells were infected with ZIKV at 37 °C for 1 h in the presence of one of the NPs, followed by removal of the unbound viruses and NPs, and culture of the cells to calculate plaques, as in (**a**). (**e**) Blockage of ZIKV penetration (membrane fusion): cells were incubated with ZIKV at 4 °C for 1 h to allow ZIKV attachment. After removal of the unbound ZIKV, the cells were incubated with one of the NPs at 37 °C for 1 h to allow fusion of virus–cell membranes. After further removal of the unbound NPs, the cells were cultured and plaques were calculated as in (**a**). (**f**) Inhibition of postentry stage: cells were incubated with ZIKV at 37 °C for 1 h to allow ZIKV entry into the target cells. After removal of the unbound ZIKV, the cells were further incubated with one of the NPs at 37 °C for 1 h, followed by removal of the unbound NPs and culture of cells for calculation of plaques, as in (**a**). Inhibition of natural products, including gossypol (**B**), curcumin (**C**), digitonin (**D**), and conessine (**E**), against ZIKV (PAN2016) infection in the six steps mentioned above. (**F**) A potent anti-ZIKV inhibitor, temoporfin [28], was used as a control for step a. (**G**) An anti-ZIKV entry (especially in inhibition of the internalization/fusion step) inhibitor, 25-hydroxycholesterol [29], was used as a control for steps b and e. Anti-ZIKV compound bortezomib was used as a control for step d (**H**), and a replication inhibitor, NITD008 [30], for step f (**I**). The natural product curcumin (**C**), which has been previously reported to inhibit the attachment of ZIKV to host cells [37], was used as a control for stage c. The percent inhibition was calculated in the presence or absence of serially diluted natural products. The data are expressed as mean ± s.e.m. (*n* = 2). The experiments were performed in Vero E6 cells and repeated three times, with similar results.

To identify which steps of ZIKV infection in its life cycle were blocked by these natural products, we carried out a time-of-addition experiment by incubating natural products with ZIKV or cells at different time points during ZIKV and cell interaction, and then calculated the percent inhibition based on the number of plaques formed [21,22,27,38]. To test whether a natural product can neutralize ZIKV infection or inhibit viral entry by targeting the viral proteins, ZIKV was pretreated with the natural product at 37 °C before incubation with the host cells (Figure 3A(a)). To evaluate whether a natural product can bind to the cellular receptors or cofactors to block virus–receptor binding, cells were pretreated with the natural product at 37 °C before incubation with ZIKV (Figure 3A(b)). To determine whether a natural product can inhibit attachment of ZIKV to target cells, but cannot block the virus–cell membrane fusion, cells were cotreated with ZIKV at 4 °C in the presence of the natural product (Figure 3A(c)). To assess whether a natural product can inhibit attachment of ZIKV to target cells and subsequent virus–cell membrane fusion, the cells were cotreated with ZIKV and the natural product at 37 °C (Figure 3A(d)). To investigate whether a natural product can inhibit ZIKV fusion with the cell membrane and then entry into the cell, cells were pretreated with ZIKV at 4 °C first and then incubated with the natural product at 37 °C (Figure 3A(e)). To study whether a natural product can inhibit ZIKV infection at postentry stages (i.e., viral replication, virion assembly, or release), cells were pretreated with ZIKV and then incubated with the natural product at 37 °C (Figure 3A(f)).

After completing the above approaches, we gained insight into the potential mechanisms of the natural products responsible for inhibiting ZIKV (PAN2016) infection. After pretreatment of ZIKV with gossypol at 37 °C before incubation with the target cells, ZIKV completely lost its infectivity, whereas it maintained its infectivity after other treatments described above (Figure 3B), suggesting that gossypol can effectively neutralize ZIKV infection by targeting the virus, rather than the cell or cell-associated entry or replication stages. The results from curcumin revealed that about 75–100% of ZIKV infection was blocked when curcumin was incubated with ZIKV only at 37 °C, or coincubated with ZIKV and cells at 4 °C or 37 °C, whereas there was low to no impact on ZIKV infection when curcumin was pretreated with cells or postincubated with ZIKV-treated cells at 4 °C and 37 °C, respectively (Figure 3C). These results suggest that curcumin inhibits ZIKV infection at the early stages of viral entry, particularly the viral attachment stage. Pretreatment of Vero E6 cells with digitonin and then with ZIKV or cotreatment of cells with ZIKV and digitonin at 37 °C significantly (≥94%) blocked ZIKV infection, whereas preincubation of cells with ZIKV and then with digitonin at 37 °C, pretreatment of cells with ZIKV at 4 °C and then with digitonin at 37 °C, or cotreatment of cells with digitonin and ZIKV at 4 °C inhibited about 49–74% of ZIKV infection (Figure 3D). In contrast, preincubation of digitonin and ZIKV had no effect on ZIKV infection (Figure 3D). These results suggest that digitonin could not directly neutralize ZIKV infection, but inhibited ZIKV infection by binding to the viral receptors or inhibiting viral entry (i.e., attachment and membrane fusion or postentry steps). The data from conessine indicated that coincubation of cells with conessine and ZIKV at 37 °C or postincubation of conessine with ZIKV-treated cells at 4 °C or 37 °C resulted in 80–96% inhibition of ZIKV infection, whereas pretreatment of cells with conessine before ZIKV incubation blocked about 38% of ZIKV infection. Nevertheless, preincubation of conessine and ZIKV at 37 °C or cotreatment of cells with conessine and ZIKV at 4 °C had very low to no effect on ZIKV infection (Figure 3E). These data suggest that conessine does not block ZIKV attachment to the host cell, but inhibits ZIKV infection by targeting virus–cell fusion or postentry step. The above steps of inhibition of ZIKV infection were further proven by the respective anti-ZIKV compound controls (Figure 3F–I). Therefore, the above data confirm the potent inhibitory activity of the identified natural products, particularly gossypol, in blocking ZIKV infection at various stages of the viral life cycle.

### 3.3. Identification of Potential Binding Regions of Lead Natural Products in ZIKV Proteins

**Figure 4:**
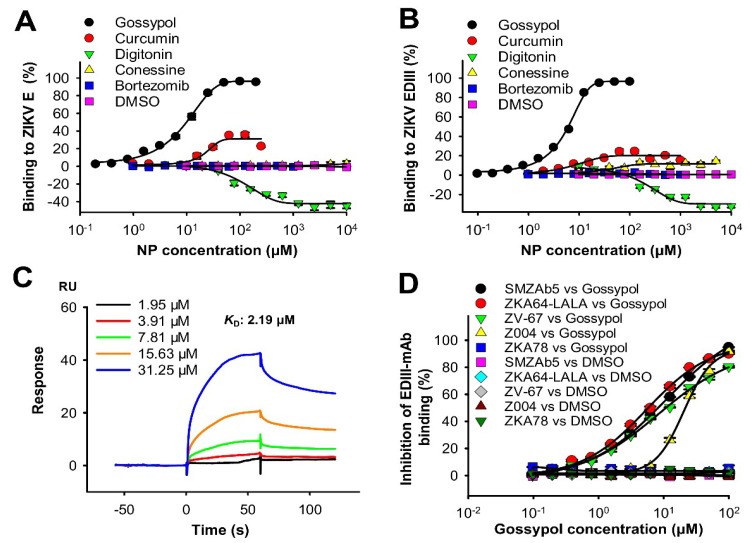
Characterization of natural products in binding to ZIKV E protein and inhibition of binding of ZIKV envelope protein domain III (EDIII)-specific mAb to EDIII. Binding of natural products (NPs) to ZIKV full-length E (**A**) or EDIII (**B**) proteins, as detected by ELISA. The percent binding was reported in the presence or absence of serially diluted NPs using the formula ((1—(E/EDIII-NP)/(E/EDIII)) × 100) for E/EDIII binding. The 50% effective concentration (EC_50_) values were calculated. The data are expressed as mean ± s.e.m. (*n* = 4). (**C**) Surface plasmon resonance (SPR) analysis of binding between gossypol and ZIKV E. Binding affinity (*K*_D_: equilibrium dissociation constant) is shown. (**D**) The ability of gossypol to inhibit the binding between ZIKV EDIII and EDIII-specific neutralizing mAbs. The concentrations of ZIKV EDIII and mAbs were 1.5 and 0.5 µg/mL, respectively. The percent inhibition in the EDIII-mAb binding was measured in the presence or absence of serially diluted gossypol using the formula ((1-(EDIII-mAb-gossypol)/(EDIII-mAb) × 100), which, in turn, formed the basis for calculating 50% inhibitory concentration (IC_50_) values. ZIKV EDI/DII-specific mAb (ZKA78) and DMSO were used as controls. The data are expressed as mean ± s.e.m. (*n* = 4). The experiments were repeated twice, with similar results.

To identify the potential binding regions of the natural products in ZIKV proteins, we first carried out an ELISA-based approach by coating the plates with ZIKV full-length E or EDIII proteins. We then tested for binding affinity using a ZIKV EDIII-specific mAb, ZKA64-LALA, for E or EDIII binding. Results revealed that gossypol bound potently to the full-length E and EDIII proteins, with EC_50_ values of 7.12 and 4.22 µM, respectively, whereas curcumin had much lower binding affinity to ZIKV full-length E and EDIII proteins (Figure 4A,B). Otherwise, digitonin, conessine, bortezomib (anti-ZIKV compound control), and DMSO (negative control) did not bind to any ZIKV proteins tested (Figure 4A,B). We then evaluated the binding of gossypol using an SPR assay, and the result showed that it had binding affinity values of 2.19 µM to ZIKV E protein (Figure 4C).

Since gossypol bound to ZIKV E protein, potentially in the EDIII region, we further carried out an ELISA completion assay to identify its possible binding site(s) in the EDIII. Accordingly, ZIKV EDIII protein was coated on the plates, and binding between ZIKV EDIII and EDIII-specific mAbs (SMZAb5, ZKA64-LALA, ZV-67, or Z004), or a ZIKV EDI/DII-specific mAb control (ZKA78) was evaluated in the presence of serially diluted gossypol. The results showed that gossypol potently blocked binding of EDIII-specific mAbs SMZAb5, ZKA64-LALA, ZV-67, or Z004 to EDIII protein in a dose-dependent manner, with IC_50_ values of 7.32, 5.72, 11.7, and 22.2 µM, respectively, whereas the DMSO control showed no blockage of this binding (Figure 4D). In the meantime, there was no binding between the control mAb ZKA78 and EDIII protein, so no obvious inhibition of gossypol was detected (Figure 4D). The above ZIKV EDIII-specific mAbs have been previously shown to potently neutralize ZIKV infection [17] and recognize epitopes, including the lateral ridge, such as residues 309–314, 331–337, 368, 370, 371, and 393–397 of ZIKV EDIII protein [39,40,41]. Therefore, the above data suggest that gossypol most likely binds to the lateral ridge of the ZIKV EDIII protein to block the EDIII-mAb binding.

The data described here demonstrate that the identified natural product gossypol bound strongly to ZIKV E protein, potentially the conserved EDIII, thus blocking EDIII-mAb binding at important neutralizing epitopes and inhibiting viral entry into the target cell. These data reasonably explain the potent broad-spectrum antiviral activity of gossypol against infections of multiple ZIKV strains.

### 3.4. Combinatorial Effects of the Combinations of Gossypol with Other Natural Products against ZIKV Infection and Their Cytotoxicity to Vero E6 Cells

Since gossypol demonstrated the highest antiviral activity individually against all ZIKV strains tested, we next investigated the potential combinatorial effects of the combination of gossypol with three other natural products identified, namely curcumin, digitonin, and conessine, as well as anti-ZIKV compound control (bortezomib). Results demonstrated significant combinatorial inhibitory effects against three ZIKV strains (PAN2016, FLR, and PRVABC59) tested when combining gossypol with any of these natural products, and the CI values ranged from 0.44 to 0.6 µM, 0.44 to 0.95 µM, and 0.19 to 0.3 µM for ZIKV PAN2016, FLR, and PRVABC59 strains, respectively (Table 3, Table 4 and Table 5). The combinations of gossypol with each of these natural products also resulted in the highest enhancement of anti-PRVABC59 activity among the three ZIKV strains tested (Table 5). These data show that gossypol can be combined with other inhibitors described above to further increase overall inhibitory activity against current and future emergent ZIKV strains.

The above results on the combinatorial effects against ZIKV infection could not exclude the possibility that the observed decrease in IC_50_ in the presence of another natural product might result from the enhanced cell death caused by the natural product in combination. Therefore, we also assessed the change of CC_50_ of the natural products alone and in combination, and compared the enhancement of CC_50_ with that of IC_50_ against ZIKV strain PRVABC59. As shown in Table 6, the cytotoxicity of gossypol in combination with curcumin, digitonin, or conessine was not enhanced. The cytotoxicity of gossypol in combination with bortezomib was slightly increased (1.35-fold), but it was much lower than the enhancement in anti-PRVABC59 activity of gossypol (8.73-fold) in combination with bortezomib. In addition, the cytotoxicity of curcumin and bortezomib, respectively, in combination with gossypol was not enhanced. The cytotoxicity of digitonin or conessine in combination with gossypol was slightly increased by about 2.60- and 5.78-fold, respectively, while they were still lower than the enhancement in anti-PRVABC59 activity of digitonin (5.98-fold) or conessine (6.29-fold) in combination with gossypol. Moreover, the CI values of gossypol with any of the four natural products tested were greater than 1 (Table 6), indicating that there was no combinatorial effect on the cytotoxicity to the test cells. These results suggest that the observed decrease in the IC_50_ values of the natural products in combination was not due to the enhancement in cytotoxicity of these natural products.

### 3.5. Potent Inhibitory Activity of Lead Natural Products, Particularly Gossypol, against Infections of DENV-1–4 Strains

Identification of broad-spectrum anti-flavivirus inhibitors is crucial to treat infections caused by ZIKV and other flaviviruses, such as DENV. Hence, using a plaque assay similar to ZIKV, we evaluated the antiviral activity of natural products against infections of four serotypes of DENV human strains in LLC-MK2 cells. Even though all four natural products could inhibit DENV-1–4 infections, results showed that gossypol had the highest potency against DENV-1, DENV-2, and DENV-4 infections, with IC_50_ values of 1.87, 1.89, and 2.6 µM, respectively (Table 7). Also, the anti-DENV-3 activity of gossypol (IC_50_ value: 3.7 µM) was only slightly higher than that of curcumin (IC_50_ value: 2.09 µM) (Table 7). The cytotoxicity of these natural products on LLC-MK2 cells was investigated by a cytotoxicity assay, with the CC_50_ values ranging from 14.54 to 302.69 µM (Table 7). The above data indicate the potent anti-DENV activity of the natural products, particularly gossypol, against infections of four DENV human strains with low to no cytotoxicity.

As described earlier, gossypol targeted ZIKV E protein, potentially EDIII. Although a number of variations have been identified in the amino acid sequences of E proteins of ZIKV and DENV strains tested in this study (Figure S3), gossypol could still inhibit all ZIKV and DENV strains tested, suggesting that it potentially targeted the conserved sequences in ZIKV and DENV EDIII proteins. Our data further explain the potent, broad-spectrum activity of gossypol against infections of at least two flaviviruses, including ZIKV and DENV.

## 4. Discussion

Development of safe and effective antiviral therapeutics is urgently needed to treat ZIKV infections and ZIKV-caused diseases, particularly ZIKV-associated microcephaly, fetal death, or neurological diseases [7,42,43,44,45]. Here, we have identified four small-molecule-based natural products, namely gossypol, curcumin, digitonin, and conessine, with robust anti-ZIKV activities in Vero E6 cells. Among them, gossypol had the greatest potency to block infections of ZIKV for almost all 10 strains isolated from 1947 to 2016 from nine countries and three hosts, including six recent human strains associated with congenital Zika syndrome and other neurological malformations. Time-of-addition-based mechanistic studies indicated that gossypol could neutralize ZIKV infection by targeting the virus rather than the cell or cell-associated ZIKV entry or replication steps. Inhibition assays revealed that gossypol strongly bound to ZIKV E protein, particularly the conserved EDIII, and blocked the binding between ZIKV EDIII and EDIII-specific neutralizing mAbs, rationalizing its efficacious and broad-spectrum anti-ZIKV activity. It appears that the binding between gossypol and ZIKV E/EDIII protein might be nonspecific, since gossypol is also active against other enveloped viruses, including herpes simplex virus type 2, parainfluenza virus type 3, influenza virus, and HIV-1 [46,47,48,49,50]. Because of the potential toxicity and side effects of gossypol to humans [50,51,52], it might not be a good idea to use this natural product as a drug to treat human diseases, including ZIKV-associated microcephaly and other flavivirus-caused diseases. Nevertheless, the goal of this study is to identify the best active, natural product, then modify it to improve its antiviral activity and drug-like properties and reduce its toxicity. Due to the symmetric nature of the gossypol molecule, there will be better opportunity to defragment its structure to smaller drug-like molecules with higher activity and lower toxicity. Therefore, it is suggested not to use gossypol as the lead molecule, but rather the final drug. Unlike gossypol, digitonin and conessine did not neutralize ZIKV directly, but instead inhibited ZIKV infection at various stages of the life cycle, including viral attachment, membrane fusion, or postentry steps. Notably, digitonin is a glycoalkaloid saponin detergent. It is widely used as a cell membrane permeabilizing agent [53,54] and a detergent for selective solubilization of membrane proteins [55,56,57]; thus, the antiviral activity of this natural product is likely to be very nonspecific. By comparison, conessine appears to be a good candidate with a decent anti-ZIKV activity and very low cytotoxicity (higher selectivity). However, it is a steroidal alkaloid [58], which might not be ideal for further optimization. Similar to gossypol, curcumin may directly neutralize ZIKV infection, but it seems to mainly inhibit the early stage (attachment) of virus infection, the mechanism of which is similar to that seen in a previous report using different approaches [37].

Our data have also demonstrated strong in vitro ability of these natural products—gossypol in particular—against four serotypes of DENV human strains with low to no cytotoxicity, and the IC_50_ values against DENV were similar to those against ZIKV. We have found that gossypol bound to ZIKV E protein, potentially EDIII, suggesting that it may recognize highly conserved regions and sites in the E proteins of ZIKV and DENV. Previously, curcumin was shown to potentially inhibit DENV-2 infection through direct effects on viral particle production or various cellular systems [38]. We have not found studies in the literature reporting on the antiviral activity of curcumin on other serotypes of DENV or the inhibitory effects of gossypol, digitonin, and conessine on ZIKV and other flaviviruses. Thus, the present study provides a rationale for further understanding of anti-DENV and anti-ZIKV mechanisms of these natural products and identifying their potential binding sites in the viral proteins.

Except for demonstrating the individual in vitro anti-ZIKV activity of natural products identified in this study, particularly gossypol, we have confirmed important combinatorial anti-ZIKV effects of gossypol in combination with other natural products. It is interesting to note that combining gossypol with curcumin, digitonin, or conessine resulted in increased combinatorial effect against PRVABC59 strain compared to the FLR strain of ZIKV, potentially because gossypol had more potent anti-FLR activity than anti-PRVABC59 activity when tested individually. Also, such combinations enhanced the individual antiviral activity of curcumin, digitonin, and conessine against the test viruses. We have shown that the combination of gossypol and bortezomib, a licensed proteasome inhibitor previously reported inhibiting ZIKV infection [16,59], led to significant combinatorial activity against the three epidemic ZIKV human strains tested. Therefore, our study demonstrates the possibility of combining gossypol with other natural products or compounds to enhance antiviral activity.

Overall, this study has evaluated the antiviral activity of four natural products in vitro, among which three are newly identified natural products with strong anti-ZIKV ability. Future observations will be needed to evaluate the protective efficacy of these natural products individually or in combination, or using their defragmented, small drug-like molecules (in the case of gossypol) against ZIKV-caused congenital infection and fetal demise in available animal models. Modification of the structure of gossypol and identification of its derivatives with better antiviral activity, but without cytotoxicity, would be essential for developing a safe and effective anti-ZIKV agent for human use. Also, future studies to evaluate the antiviral activity of modified gossypol or its derivatives against other flaviviruses, both in vitro and in vivo, will be helpful for identification of an effective and safe pan-flavivirus inhibitor. Taken together, the broad-spectrum ability of the identified natural products, especially gossypol, against ZIKV and DENV infections indicates the potential for further development of these small molecules or their derivatives as the lead compounds or effective anti-flavivirus inhibitors.

## Figures and Tables

**Table 1:** Initial screening of a natural product library for identification of potential anti-ZIKV inhibitors.

Parameters	No. (%)
Natural products from MicroSource Discovery Systems for screening	720
Primary hits (with inhibition against ZIKV strain PAN2016) ^a^	61 (8.5)
Primary hits (with high cytotoxicity in Vero E6 cells) ^b^	38 (5.3)
Specific hits (primary hits with low cytotoxicity)	23 (3.2)
Specific hits (available to purchase)	16 (2.2)
Specific hits (ordered from Sigma and retested to confirm anti-ZIKV activity)	6 (0.8)
Specific hits displaying IC_50_ < CC_50_	4 (0.6)

^a^ A total of 61 primary hits inhibited ZIKV (strain PAN2016) infection by more than 70% at 20 μM, whereas the negative control (DMSO) had inhibitory activity less than 10%. ^b^ Observed cytotoxicity of natural products (at 20 μM) under a microscope. The cytotoxicity was recorded as 6 grades (−, ±, +, ++, +++, ++++), and the natural products with cytotoxicity greater than or equal to grade ++ were referred to as “high cytotoxicity in Vero E6 cells”, and discarded for further testing.

**Table 2:** Inhibitory activity of natural products against infections of ZIKV with different strains.

Natural			IC_50_ (μM)								
Products	CC_50_ (μM)	PAN2016	R116265	PAN2015	FLR	R103451	PRVABC59	PLCal_ZV	IbH 30656	MEX 2–81	MR 766
Gossypol	14.17 ± 0.74	3.48 ± 0.03	4.20 ± 0.08	3.95 ± 0.05	0.21 ± 0.01	2.28 ± 0.10	4.31 ± 0.02	1.98 ± 0.07	3.31 ± 0.11	2.79 ± 0.01	3.75 ± 0.01
Curcumin	52.86 ± 0.52	13.67 ± 0.72	14.04 ± 0.15	13.71 ± 0.37	16.57 ± 0.34	11.22 ± 0.37	12.85 ± 0.35	10.84 ± 0.73	13.63 ± 0.31	5.62 ± 0.52	11.42 ± 0.29
Digitonin	56.29 ± 1.20	4.31 ± 0.23	6.52 ± 0.59	5.00 ± 0.01	3.34 ± 0.22	4.30 ± 0.43	3.76 ± 1.12	3.19 ± 0.25	5.5.30 ± 0.13	3.84 ± 0.12	3.77 ± 0.31
Conessine	323.71 ± 0.25	9.75 ± 0.26	7.18 ± 0.13	7.98 ± 0.29	9.65 ± 0.58	11.60 ± 0.33	9.08 ± 0.33	8.11 ± 0.37	10.25 ± 0.41	10.94 ± 0.06	7.44 ± 0.11
Bortezomib	16.96 ± 0.20	9.75 ± 0.03	8.94 ± 0.10	9.88 ± 0.12	9.62 ± 0.59	14.14 ± 0.85	11.72 ± 0.82	31.04 ± 0.71	9.35 ± 0.23	7.67 ± 0.31	9.51 ± 0.26

Note: The experiments were performed on Vero E6 cells, and the cytotoxicity of natural products in this cell line is expressed as 50% cytotoxic concentration (CC_50_). The inhibitory activity of natural products against ZIKV infection is expressed as a 50% inhibitory concentration (IC_50_). Bortezomib was used as an anti-ZIKV compound control. The data are expressed as the mean ± standard error of the mean (s.e.m.) (*n* = 2). The experiments were repeated twice, with similar results.

**Table 3:** Combinatorial effects of gossypol with other natural products in inhibition of infection of ZIKV PAN2016 strain.

Natural Product	IC_50_ (μM)		Fold of Enhancement	Natural Products	IC_50_ (μM)		Fold of Enhancement	CI
	Alone	In Mixture			Alone	In Mixture		
Gossypol	3.79 ± 0.01	0.93 ± 0.04	3.08	Curcumin	13.20 ± 0.81	3.67 ± 0.18	2.60	0.52
	3.79 ± 0.01	1.08 ± 0.19	2.51	Digitonin	4.85 ± 0.24	1.51 ± 0.27	2.21	0.60
	3.79 ± 0.01	0.81 ± 0.11	3.68	Conessine	10.04 ± 0.25	2.26 ± 0.30	3.44	0.44
	3.79 ± 0.01	1.00 ± 0.02	2.79	Bortezomib	10.65 ± 0.01	2.79 ± 0.06	2.82	0.53

Note: The experiments were performed on Vero E6 cells, and the inhibitory activity of natural products against infection of ZIKV (strain PAN2016) is expressed as IC_50_. Ratios of molar concentrations of gossypol and curcumin, digitonin, conessine (three lead natural products) or bortezomib (anti-ZIKV compound control) in combination against ZIKV strain PAN2016 (2.5 × 10^3^ PFU; MOI ~0.025) are 0.29:1, 0.78:1, 0.38:1, and 0.36:1, respectively. Fold of enhancement was calculated using the formula ((IC_50_ alone)/(IC_50_ in the mixture)—1). Combination index (CI) was calculated using the formula ((IC_50_ in the mixture)1/(IC_50_ alone)1 + (IC_50_ in the mixture)2/(IC_50_ alone)2), where 1 and 2 represent two natural products in the combination, respectively. The data are expressed as mean ± s.e.m. (*n* = 2). The experiments were repeated twice, with similar results.

**Table 4:** Combinatorial effects of gossypol with other natural products in inhibition of infection of ZIKV FLR strain.

Natural Product	IC_50_ (μM)		Fold of Enhancement	Natural Products	IC_50_ (μM)		Fold of Enhancement	CI
	Alone	In Mixture			Alone	In Mixture		
Gossypol	0.26 ± 0.01	0.06 ± 0.01	3.33	Curcumin	17.05 ± 0.08	4.44 ± 0.74	2.84	0.49
	0.26 ± 0.01	0.12 ± 0.01	1.17	Digitonin	3.86 ± 0.02	1.89 ± 0.13	12.04	0.95
	0.26 ± 0.01	0.10 ± 0.01	2.60	Conessine	10.07 ± 0.45	4.73 ± 0.08	1.13	0.85
	0.26 ± 0.01	0.05 ± 0.01	4.20	Bortezomib	9.70 ± 0.76	2.40 ± 0.28	3.04	0.44

Note: The experiments were performed on Vero E6 cells, and the inhibitory activity of natural products against infection of ZIKV (strain FLR) is expressed as IC_50_. Ratios of molar concentrations of gossypol and curcumin, digitonin, conessine (three lead natural products) or bortezomib (anti-ZIKV compound control) in combination against ZIKV strain FLR (2.5 × 10^3^ PFU; MOI ~0.025) are 0.02:1, 0.07:1, 0.03:1, and 0.03:1, respectively. Fold of enhancement was calculated using the formula ((IC_50_ alone)/(IC_50_ in the mixture)—1). Combination index (CI) was calculated using the formula ((IC_50_ in the mixture)1/(IC_50_ alone)1 + (IC_50_ in the mixture)2/(IC_50_ alone)2), where 1 and 2 represent two natural products in the combination, respectively. The data are expressed as mean ± s.e.m. (*n* = 2). The experiments were repeated twice, with similar results.

**Table 5:** Combinatorial effects of gossypol with other natural products in inhibition of infection of ZIKV PRVABC59 strain.

Natural Product	IC_50_ (μM)		Fold of Enhancement	Natural Products	IC_50_ (μM)		Fold of Enhancement	CI
	Alone	In Mixture			Alone	In Mixture		
Gossypol	4.38 ± 0.08	0.65 ± 0.06	5.74	Curcumin	12.46 ± 0.05	1.93 ± 0.16	5.46	0.30
	4.38 ± 0.08	0.63 ± 0.10	5.95	Digitonin	3.84 ± 0.81	0.55 ± 0.09	5.98	0.29
	4.38 ± 0.08	0.62 ± 0.01	6.06	Conessine	9.40 ± 0.21	1.29 ± 0.01	6.29	0.28
	4.38 ± 0.08	0.45 ± 0.02	8.73	Bortezomib	12.17 ± 0.07	1.07 ± 0.04	10.37	0.19

Note: The experiments were performed on Vero E6 cells, and the inhibitory activity of natural products against infection of ZIKV (strain PRVABC59) is expressed as IC_50_. Ratios of molar concentrations of gossypol and curcumin, digitonin, conessine (three lead natural products) or bortezomib (anti-ZIKV compound control) in combination against ZIKV strain PRVABC59 (2.5 × 10^3^ PFU; MOI ~0.025) are 0.35:1, 1.14:1, 0.47:1, and 0.36:1, respectively. Fold of enhancement was calculated using the formula ((IC_50_ alone)/(IC_50_ in the mixture)—1). Combination index (CI) was calculated using the formula ((IC_50_ in the mixture)1/(IC_50_ alone)1 + (IC_50_ in the mixture)2/(IC_50_ alone)2), where 1 and 2 represent two natural products in the combination, respectively. The data are expressed as mean ± s.e.m. (*n* = 2). The experiments were repeated twice, with similar results.

**Table 6:** Potential cytotoxicity of gossypol in combination with other natural products to Vero E6 cells.

Natural Product	CC_50_ (μM)		Fold of Enhancement	Natural Products	CC_50_ (μM)		Fold of Enhancement	CI
	Alone	In Mixture			Alone	In Mixture		
Gossypol	14.84 ± 0.42	13.70 ± 0.05	0.08	Curcumin	53.12 ± 1.83	41.36± 1.93	0.28	1.70
	14.84 ± 0.42	16.01 ± 0.40	‒0.07	Digitonin	50.63 ± 0.22	14.05 ± 0.35	2.60	1.36
	14.84 ± 0.42	15.16 ± 0.80	‒0.02	Conessine	314.57 ± 2.32	46.39 ± 2.94	5.78	1.17
	14.84 ± 0.42	6.31 ± 0.62	1.35	Bortezomib	17.77 ± 0.17	17.53 ± 1.73	0.01	1.41

Note: Gossypol with one of the natural products, including curcumin, digitonin, conessine (three lead natural products) or bortezomib (anti-ZIKV compound control), were first mixed according to the molar ratio identified in the above combinational experiment against ZIKV strain PRVABC59, and then added to Vero E6 cells to determine cytotoxicity. The combinatorial cytotoxicity of natural products to Vero E6 cells is expressed as CC_50_ in the mixture. Ratios of molar concentrations of gossypol and curcumin, digitonin, conessine, or bortezomib in combination are 0.35:1, 1.14:1, 0.47:1, and 0.36:1, respectively. Fold of enhancement was calculated using the formula ((CC_50_ alone)/(CC_50_ in the mixture)—1). Combination index (CI) was calculated using the formula ((CC_50_ in the mixture)1/(CC_50_ alone)1 + (CC_50_ in the mixture)2/(CC_50_ alone)2), where 1 and 2 represent two natural products in the combination, respectively. The data are expressed as mean ± s.e.m. (*n* = 2). The experiments were repeated twice, with similar results.

**Table 7:** Inhibitory activity of natural products against infections of DENV-1–4.

Natural Products		IC_50_ (μM)			
	CC_50_ (μM)	DENV-1-V1792	DENV-2-V594	DENV-3-V1043	DENV-4-PR 06-65-740
Gossypol	14.54 ± 0.59	1.87 ± 0.01	1.89 ± 0.21	3.70 ± 0.59	2.60 ± 0.12
Curcumin	59.42 ± 1.18	9.37 ± 0.47	3.07 ± 0.07	2.09 ± 0.12	4.83 ± 0.24
Digitonin	59.02 ± 0.33	5.21 ± 0.35	6.56 ± 0.21	4.07 ± 0.83	6.44 ± 0.34
Conessine	302.69 ± 13.40	7.09 ± 0.08	6.61 ± 0.60	7.41 ± 0.04	7.27 ± 0.31

Note: The experiments were performed on LLC-MK2 cells, and the cytotoxicity of natural products in this cell line is expressed as CC_50_. The inhibitory activity of natural products against infections of DENV-1–4 is expressed as IC_50_. The data are expressed as mean ± s.e.m. (*n* = 2). The experiments were repeated twice, with similar results.

## Supplementary Materials

The following are available online at https://www.mdpi.com/1999-4915/11/11/1019/s1. Figure S1: Association between inhibitory activity of natural products against ZIKV strain PAN2016 and their cytotoxicity. Figure S2: Association between inhibitory activity of natural products against ZIKV strain PRVABC59 and their cytotoxicity. Figure S3: Multiple sequence alignment of amino acid (aa) sequences of E protein of 10 ZIKV strains and DENV-1-3 human strains used in this study.

## References

[1] Lazear H.M., Diamond M.S. (2016). Zika virus: New clinical syndromes and its emergence in the Western Hemisphere. J. Virol..

[2] Dick G.W., Kitchen S.F., Haddow A.J. (1952). Zika virus (I). Isolations and serological specificity. Trans. R. Soc. Trop. Med. Hyg..

[3] Zorrilla C.D., Garcia Garcia I., Garcia Fragoso L., De La Vega A. (2017). Zika virus infection in pregnancy: Maternal, fetal, and neonatal considerations. J. Infect. Dis..

[4] Castro M.C., Han Q.C., Carvalho L.R., Victora C.G., Franca G.V.A. (2018). Implications of Zika virus and congenital Zika syndrome for the number of live births in Brazil. Proc. Natl. Acad. Sci. USA.

[5] Lucey D., Cummins H., Sholts S. (2017). Congenital Zika syndrome in 2017. JAMA.

[6] Driggers R.W., Ho C.Y., Korhonen E.M., Kuivanen S., Jaaskelainen A.J., Smura T., Rosenberg A., Hill D.A., DeBiasi R.L., Vezina G. (2016). Zika virus infection with prolonged maternal viremia and fetal brain abnormalities. N. Engl. J. Med..

[7] Brasil P., Pereira J.P., Moreira M.E., Ribeiro Nogueira R.M., Damasceno L., Wakimoto M., Rabello R.S., Valderramos S.G., Halai U.A., Salles T.S. (2016). Zika virus infection in pregnant women in Rio de Janeiro. N. Engl. J. Med..

[8] Kuno G., Chang G.J. (2007). Full-length sequencing and genomic characterization of Bagaza, Kedougou, and Zika viruses. Arch. Virol..

[9] Agrelli A., de Moura R.R., Crovella S., Brandao L.A.C. (2019). ZIKA virus entry mechanisms in human cells. Infect. Genet. Evol..

[10] Abrams R.P.M., Solis J., Nath A. (2017). Therapeutic approaches for Zika virus infection of the nervous system. Neurotherapeutics.

[11] Lian W., Jang J., Potisopon S., Li P.C., Rahmeh A., Wang J., Kwiatkowski N.P., Gray N.S., Yang P.L. (2018). Discovery of immunologically inspired small molecules that target the viral envelope protein. ACS Infect. Dis..

[12] De Wispelaere M., Lian W., Potisopon S., Li P.C., Jang J., Ficarro S.B., Clark M.J., Zhu X., Kaplan J.B., Pitts J.D. (2018). Inhibition of flaviviruses by targeting a conserved pocket on the viral envelope protein. Cell Chem. Biol..

[13] Culshaw A., Mongkolsapaya J., Screaton G.R. (2017). The immunopathology of dengue and Zika virus infections. Curr. Opin. Immunol..

[14] Guzman M.G., Halstead S.B., Artsob H., Buchy P., Farrar J., Gubler D.J., Hunsperger E., Kroeger A., Margolis H.S., Martinez E. (2010). Dengue: A continuing global threat. Nat. Rev. Microbiol..

[15] Tai W., He L., Wang Y., Sun S., Zhao G., Luo C., Li P., Zhao H., Fremont D.H., Li F. (2018). Critical neutralizing fragment of Zika virus EDIII elicits cross-neutralization and protection against divergent Zika viruses. Emerg. Microbes Infect..

[16] Barrows N.J., Campos R.K., Powell S.T., Prasanth K.R., Schott-Lerner G., Soto-Acosta R., Galarza-Munoz G., McGrath E.L., Urrabaz-Garza R., Gao J. (2016). A screen of FDA-approved drugs for inhibitors of Zika virus infection. Cell Host Microbe.

[17] Tai W., Chen J., Zhao G., Geng Q., He L., Chen Y., Zhou Y., Li F., Du L. (2019). Rational design of Zika virus subunit vaccine with enhanced efficacy. J. Virol..

[18] Tai W., Voronin D., Chen J., Bao W., Kessler D.A., Shaz B., Jiang S., Yazdanbakhsh K., Du L. (2019). Transfusion-transmitted Zika virus infection in pregnant mice leads to broad tissue tropism with severe placental damage and fetal demise. Front. Microbiol..

[19] Jiang S., Lu H., Liu S., Zhao Q., He Y., Debnath A.K. (2004). N-substituted pyrrole derivatives as novel human immunodeficiency virus type 1 entry inhibitors that interfere with the gp41 six-helix bundle formation and block virus fusion. Antimicrob. Agents Chemother..

[20] Chou T.C. (2006). Theoretical basis, experimental design, and computerized simulation of synergism and antagonism in drug combination studies. Pharmacol. Rev..

[21] Si L., Meng K., Tian Z., Sun J., Li H., Zhang Z., Soloveva V., Li H., Fu G., Xia Q. (2018). Triterpenoids manipulate a broad range of virus-host fusion via wrapping the HR2 domain prevalent in viral envelopes. Sci. Adv..

[22] Chen M., Aoki-Utsubo C., Kameoka M., Deng L., Terada Y., Kamitani W., Sato K., Koyanagi Y., Hijikata M., Shindo K. (2017). Broad-spectrum antiviral agents: Secreted phospholipase A_2_ targets viral envelope lipid bilayers derived from the endoplasmic reticulum membrane. Sci. Rep..

[23] Basu A., Li B., Mills D.M., Panchal R.G., Cardinale S.C., Butler M.M., Peet N.P., Majgier-Baranowska H., Williams J.D., Patel I. (2011). Identification of a small-molecule entry inhibitor for filoviruses. J. Virol..

[24] Yu M., Si L., Wang Y., Wu Y., Yu F., Jiao P., Shi Y., Wang H., Xiao S., Fu G. (2014). Discovery of pentacyclic triterpenoids as potential entry inhibitors of influenza viruses. J. Med. Chem..

[25] Lu L., Pan C., Li Y., Lu H., He W., Jiang S. (2012). A bivalent recombinant protein inactivates HIV-1 by targeting the gp41 prehairpin fusion intermediate induced by CD4 D1D2 domains. Retrovirology.

[26] Yu Y., Deng Y.Q., Zou P., Wang Q., Dai Y., Yu F., Du L., Zhang N.N., Tian M., Hao J.N. (2017). A peptide-based viral inactivator inhibits Zika virus infection in pregnant mice and fetuses. Nat. Commun..

[27] Aoki-Utsubo C., Chen M., Hotta H.J.B.-P. (2018). Time-of-addition and temperature-shift assays to determine particular step(s) in the viral life cycle that is blocked by antiviral substance(s). Bio-Protocol.

[28] Li Z., Brecher M., Deng Y.-Q., Zhang J., Sakamuru S., Liu B., Huang R., Koetzner C.A., Allen C.A., Jones S.A. (2017). Existing drugs as broad-spectrum and potent inhibitors for Zika virus by targeting NS2B-NS3 interaction. Cell Res..

[29] Li C., Deng Y.Q., Wang S., Ma F., Aliyari R., Huang X.Y., Zhang N.N., Watanabe M., Dong H.L., Liu P. (2017). 25-Hydroxycholesterol protects host against Zika virus infection and its associated microcephaly in a mouse model. Immunity.

[30] Deng Y.Q., Zhang N.N., Li C.F., Tian M., Hao J.N., Xie X.P., Shi P.Y., Qin C.F. (2016). Adenosine analog NITD008 is a potent inhibitor of Zika virus. Open Forum. Infect. Dis..

[31] Du L., Tai W., Yang Y., Zhao G., Zhu Q., Sun S., Liu C., Tao X., Tseng C.K., Perlman S. (2016). Introduction of neutralizing immunogenicity index to the rational design of MERS coronavirus subunit vaccines. Nat. Commun..

[32] He L., Tai W., Li J., Chen Y., Gao Y., Li J., Sun S., Zhou Y., Du L., Zhao G. (2019). Enhanced ability of oligomeric nanobodies targeting MERS coronavirus receptor-binding domain. Viruses.

[33] Stettler K., Beltramello M., Espinosa D.A., Graham V., Cassotta A., Bianchi S., Vanzetta F., Minola A., Jaconi S., Mele F. (2016). Specificity, cross-reactivity, and function of antibodies elicited by Zika virus infection. Science.

[34] Zhao G., He L., Sun S., Qiu H., Tai W., Chen J., Li J., Chen Y., Guo Y., Wang Y. (2018). A novel nanobody targeting Middle East respiratory syndrome coronavirus (MERS-CoV) receptor-binding domain has potent cross-neutralizing activity and protective efficacy against MERS-CoV. J. Virol..

[35] Qi Q., Wang Q., Chen W., Yu F., Du L., Dimitrov D.S., Lu L., Jiang S. (2017). Anti-HIV antibody and drug combinations exhibit synergistic activity against drug-resistant HIV-1 strains. J. Infect..

[36] Wang C., Hua C., Xia S., Li W., Lu L., Jiang S. (2019). Combining a fusion inhibitory peptide targeting the MERS-CoV S2 protein HR1 domain and a neutralizing antibody specific for the S1 protein receptor-binding domain (RBD) showed potent synergism against pseudotyped MERS-CoV with or without mutations in RBD. Viruses.

[37] Mounce B.C., Cesaro T., Carrau L., Vallet T., Vignuzzi M. (2017). Curcumin inhibits Zika and chikungunya virus infection by inhibiting cell binding. Antivir. Res..

[38] Padilla S.L., Rodriguez A., Gonzales M.M., Gallego G.J., Castano O.J. (2014). Inhibitory effects of curcumin on dengue virus type 2-infected cells in vitro. Arch. Virol..

[39] Ravichandran S., Hahn M., Belaunzarán-Zamudio P.F., Ramos-Castañeda J., Nájera-Cancino G., Caballero-Sosa S., Navarro-Fuentes K.R., Ruiz-Palacios G., Golding H., Beigel J.H. (2019). Differential human antibody repertoires following Zika infection and the implications for serodiagnostics and disease outcome. Nat. Commun..

[40] Zhao H., Fernandez E., Dowd K.A., Speer S.D., Platt D.J., Gorman M.J., Govero J., Nelson C.A., Pierson T.C., Diamond M.S. (2016). Structural basis of Zika virus-specific antibody protection. Cell.

[41] Robbiani D.F., Bozzacco L., Keeffe J.R., Khouri R., Olsen P.C., Gazumyan A., Schaefer-Babajew D., Avila-Rios S., Nogueira L., Patel R. (2017). Recurrent potent human neutralizing antibodies to Zika virus in Brazil and Mexico. Cell.

[42] Cauchemez S., Besnard M., Bompard P., Dub T., Guillemette-Artur P., Eyrolle-Guignot D., Salje H., Van Kerkhove M.D., Abadie V., Garel C.J.T.L. (2016). Association between Zika virus and microcephaly in French Polynesia, 2013–2015: A retrospective study. Lancet.

[43] De Oliveira W.K., de Franca G.V.A., Carmo E.H., Duncan B.B., de Souza Kuchenbecker R., Schmidt M.I. (2017). Infection-related microcephaly after the 2015 and 2016 Zika virus outbreaks in Brazil: A surveillance-based analysis. Lancet.

[44] Krauer F., Riesen M., Reveiz L., Oladapo O.T., Martinez-Vega R., Porgo T.V., Haefliger A., Broutet N.J., Low N., WHO Zika Causality Working Group (2017). Zika virus infection as a cause of congenital brain abnormalities and Guillain–Barré syndrome: Systematic review. PLoS Med..

[45] Salinas J.L., Walteros D.M., Styczynski A., Garzón F., Quijada H., Bravo E., Chaparro P., Madero J., Acosta-Reyes J., Ledermann J. (2017). Zika virus disease-associated Guillain-Barré syndrome—Barranquilla, Colombia 2015–2016. J. Neurol. Sci..

[46] Dorsett P.H., Kerstine E.E., Powers L.J. (1975). Letter: Antiviral activity of gossypol and apogossypol. J. Pharm. Sci..

[47] Royer R.E., Deck L.M., Vander Jagt T.J., Martinez F.J., Mills R.G., Young S.A., Vander Jagt D.L. (1995). Synthesis and anti-HIV activity of 1,1′-dideoxygossypol and related compounds. J. Med. Chem..

[48] Goriunova L.V., Vichkanova S.A. (1969). Study of antiviral effect of gossypol on chick embryo model. Farmakol. Toksikol..

[49] Lin T.S., Schinazi R., Griffith B., August E., Eriksson B., Zheng D., Huang L., Prusoff W. (1989). Selective inhibition of human immunodeficiency virus type 1 replication by the (-) but not the (+) enantiomer of gossypol. Antimicrob. Agents Chemother..

[50] Dodou K., Anderson R.J., Small D.A., Groundwater P.W. (2005). Investigations on gossypol: Past and present developments. Expert Opin. Investig. Drugs.

[51] Kovacic P. (2003). Mechanism of drug and toxic actions of gossypol: Focus on reactive oxygen species and electron transfer. Curr. Med. Chem..

[52] Zbidah M., Lupescu A., Shaik N., Lang F. (2012). Gossypol-induced suicidal erythrocyte death. Toxicology.

[53] Esparis-Ogando A., Zurzolo C., Rodriguez-Boulan E. (1994). Permeabilization of MDCK cells with cholesterol binding agents: Dependence on substratum and confluency. Am. J. Physiol..

[54] Keukens E.A., de Vrije T., Jansen L.A., de Boer H., Janssen M., de Kroon A.I., Jongen W.M., de Kruijff B. (1996). Glycoalkaloids selectively permeabilize cholesterol containing biomembranes. Biochim. Biophys. Acta.

[55] Xie M., Low M.G. (1995). Streptolysin-O induces release of glycosylphosphatidylinositol-anchored alkaline phosphatase from ROS cells by vesiculation independently of phospholipase action. Biochem. J..

[56] Banerjee P., Joo J.B., Buse J.T., Dawson G. (1995). Differential solubilization of lipids along with membrane proteins by different classes of detergents. Chem. Phys. Lipids.

[57] Harder T., Kellner R., Parton R.G., Gruenberg J. (1997). Specific release of membrane-bound annexin II and cortical cytoskeletal elements by sequestration of membrane cholesterol. Mol. Biol. Cell.

[58] Dua V.K., Verma G., Singh B., Rajan A., Bagai U., Agarwal D.D., Gupta N.C., Kumar S., Rastogi A. (2013). Anti-malarial property of steroidal alkaloid conessine isolated from the bark of Holarrhena antidysenterica. Malar. J..

[59] Merin N., Kelly K. (2015). Clinical use of proteasome inhibitors in the treatment of multiple myeloma. Pharmaceuticals.

