# Supplementary material for: Identification of Novel Natural Products as Effective and Broad-Spectrum Anti-Zika Virus Inhibitors
Source: Viruses. 2019 Nov 2;11(11):1019. doi: 10.3390/v11111019 (PMC6893700; doi:10.3390/v11111019)
Supplement: Supplementary file 1 [file viruses-11-01019-s001.pdf]

Supplementary materials

Figure S1

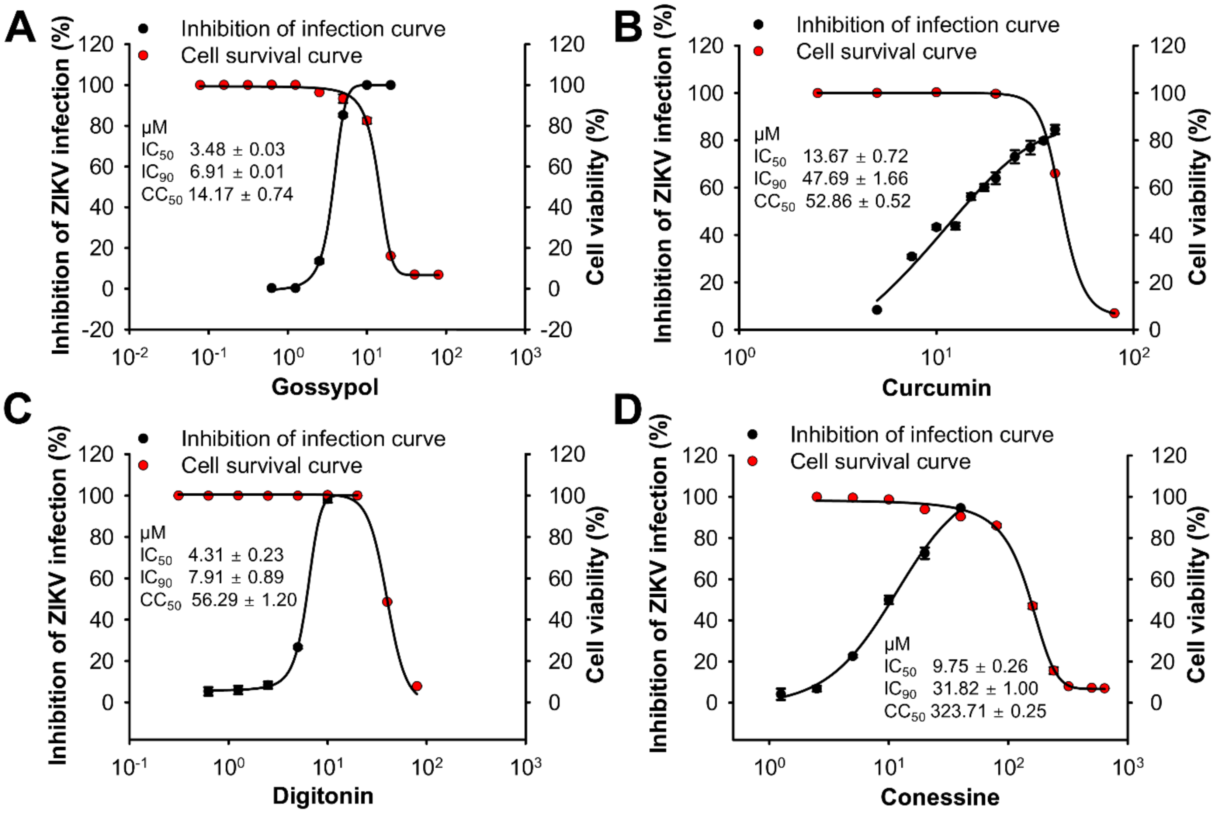

Figure S1. Association between inhibitory activity of natural products against ZIKV strain PAN2016 and their cytotoxicity. ZIKV stain PAN2016 (100 PFU) was incubated with each of the natural products at different concentrations at 37°C for 1 h. The compound-virus mixtures were then transferred to Vero E6 cells (10^5/well) and incubated at 37°C for 1 h. Plates were incubated at 37°C for 4-5 days. Viral titers at each concentration were calculated by plaque assay, which are expressed as percentage inhibition of the untreated virus (black circles). Cell viability was assessed by the Cell Counting Kit-8 (CCK8) assay, and expressed as a percentage relative to that of the untreated cells (red circles). The concentrations of natural products that inhibited 50% (IC_{50}) or 90% (IC_{90}) of plaque formation, or caused 50% cytotoxicity (CC_{50}) in Vero E6 cells are shown in the figure. The data are expressed as the mean ± standard error of the mean (s.e.m.) (n=2). The experiments were repeated twice with similar results.

Figure S2

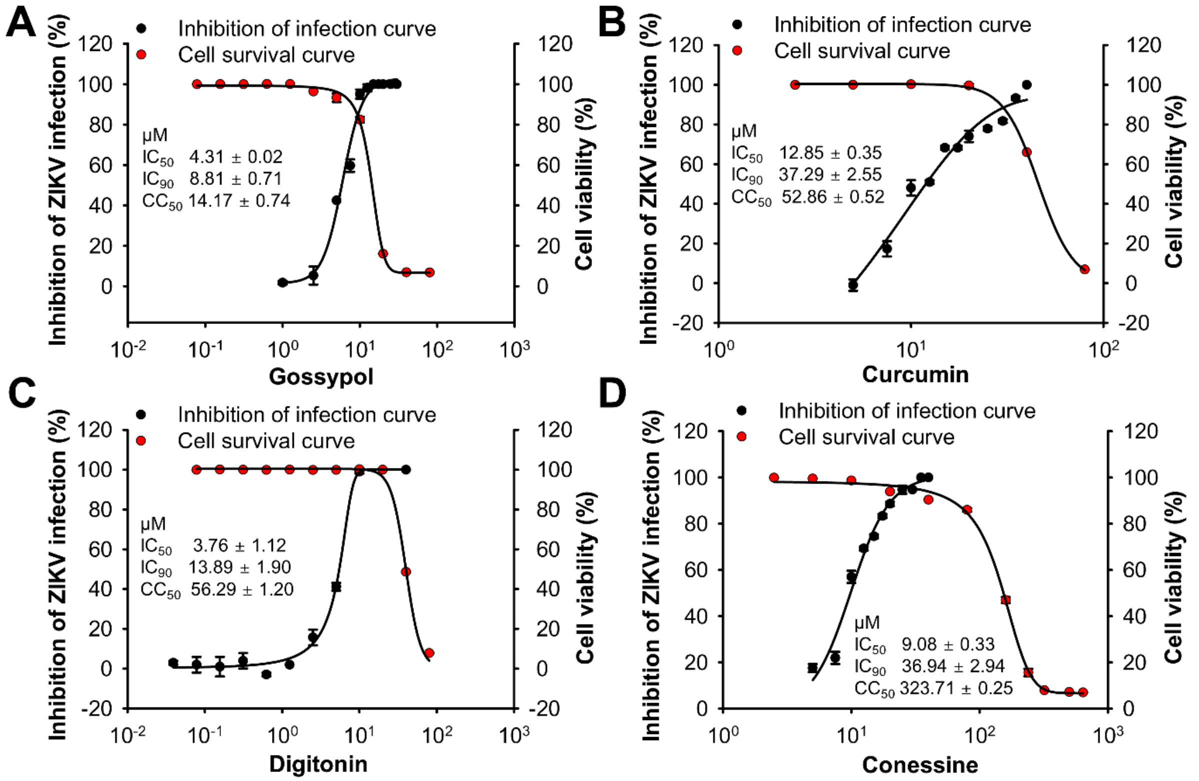

Figure S2. Association between inhibitory activity of natural products against ZIKV strain PRVABC59 and their cytotoxicity. ZIKV stain PRVABC59 (100 PFU) was incubated with each of the natural products at different concentrations at 37°C for 1 h. The compound-virus mixtures were then transferred to Vero E6 cells (10^5/well) and incubated at 37°C for 1 h. Plates were further incubated at 37°C for 4-5 days. Viral titers at each concentration were calculated by plaque assay, which are expressed as percentage inhibition of the untreated virus (black circles). Cell viability was assessed by the Cell Counting Kit-8 (CCK8) assay, and expressed as a percentage relative to that of the untreated cells (red circles). The concentrations of natural products that inhibited 50% (IC_{50}) or 90% (IC_{90}) of plaque formation, or caused 50% cytotoxicity (CC_{50}) in Vero E6 cells are shown in the figure. The data are expressed as the mean ± standard error of the mean (s.e.m.) (n=2). The experiments were repeated twice with similar results.

Figure S3

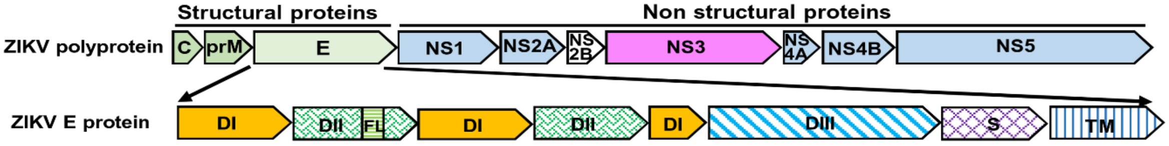


```
R116265        --IRCIGVSNRDFVEGMSGGTWVDVVLEHGGCVTVMAQDKPTVDIELVTTTVSNMAEVRS   58
PAN2016        --IRCIGVSNRDFVEGMSGGTWVDVVLEHGGCVTVMAQDKPTVDIELVTTTVSNMAEVRS   58
PAN2015        --IRCIGVSNRDFVEGMSGGTWVDVVLEHGGCVTVMAQDKPTVDIELVTTTVSNMAEVRS   58
FLR            --IRCIGVSNRDFVEGMSGGTWVDVVLEHGGCVTVMAQDKPTVDIELVTTTVSNMAEVRS   58
R103451        --IRCIGVSNRDFVEGMSGGTWVDVVLEHGGCVTVMAQDKPTVDIELVTTTVSNMAEVRS   58
PRVABC59       --IRCIGVSNRDFVEGMSGGTWVDVVLEHGGCVTVMAQDKPTVDIELVTTTVSNMAEVRS   58
PLCal_ZV       --IRCIGVSNRDFVEGMSGGTWVDVVLEHGGCVTVMAQDKPTVDIELVTTTVSNMAEVRS   58
IbH 30656      --IRCIGVSNRDFVEGMSGGTWVDVVLEHGGCVTVMAQDKPTVDIELVTTTVSNMAEVRS   58
MEX 2-81       --IRCIGVSNRDFVEGMSGGTWVDVVLEHGGCVTVMAQDKPTVDIELVTTTVSNMAEVRS   58
MR 766         --IRCIGVSNRDFVEGMSGGTWVDVVLEHGGCVTVMAQDKPTVDIELVTTTVSNMAEVRS   58
DENV-1-V1792   MAMRCVGIGSRDFVEGLSGATWVDVVLEHGSCVTTMAKDKPTLDIELLKTEVTNPAVLRK   60
DENV-2-V594    --MRCIGISNRDFVEGVSGGSWVDIVLEHGSCVTTMAKNKPTLDFELIKTEAKQPATLRK   58
DENV-3-V1043   --MRCVGVGNRDFVEGLSGATWVDVVLEHGGCVTTMAKNKPTLDIELQKTEATQLATLRK   58
```

```
R116265        YCYEASISDMASDSRCPTQGEAYLDKQSDTQYVCKRTLVDRGWGNGCGLFGKGSLVTCAK   118
PAN2016        YCYEASISDMASDSRCPTQGEAYLDKQSDTQYVCKRTLVDRGWGNGCGLFGKGSLVTCAK   118
PAN2015        YCYEASISDMASDSRCPTQGEAYLDKQSDTQYVCKRTLVDRGWGNGCGLFGKGSLVTCAK   118
FLR            YCYEASISDMASDSRCPTQGEAYLDKQSDTQYVCKRTLVDRGWGNGCGLFGKGSLVTCAK   118
R103451        YCYEASISDMASDSRCPTQGEAYLDKQSDTQYVCKRTLVDRGWGNGCGLFGKGSLVTCAK   118
PRVABC59       YCYEASISDMASDSRCPTQGEAYLDKQSDTQYVCKRTLVDRGWGNGCGLFGKGSLVTCAK   118
PLCal_ZV       YCYEASISDMASDSRCPTQGEAYLDKQSDTQYVCKRTLVDRGWGNGCGLFGKGSLVTCAK   118
IbH 30656      YCYEASISDMASDSRCPTQGEAYLDKQSDTQYVCKRTLVDRGWGNGCGLFGKGSLVTCAK   118
MEX 2-81       YCYEASISDMASDSRCPTQGEAYLDKQSDTQYVCKRTLVDRGWGNGCGLFGKGSLVTCAK   118
MR 766         YCYEASISDMASDSRCPTQGEAYLDKQSDTQYVCKRTLVDRGWGNGCGLFGKGSLVTCAK   118
DENV-1-V1792   LCIEAKISNTTTDSRCPTQGEATLVEEQDANFVCRRTFVDRGWGNGCGLFGKGSLITCAK   120
DENV-2-V594    YCIEAKLTNTTTESRCPTQGEPSLNEEQDKRFICKHSMVDRGWGNGCGLFGKGGIVTCAM   118
DENV-3-V1043   LCIEGKITNITTDSRCPTQGEAVLPEEQDQNYVCKHTYVDRGWGNGCGLFGKGSLVTCAK   118
```

```
R116265        FACSKKMTGKSIQPENLEYRIMLSVH-GSQHSGMIVNDTGHETDENRAKVEITPNSPRAE   177
PAN2016        FACSKKMTGKSIQPENLEYRIMLSVH-GSQHSGMIVNDTGHETDENRAKVEITPNSPRAE   177
PAN2015        FACSKKMTGKSIQPENLEYRIMLSVH-GSQHSGMIVNDTGHETDENRAKVEITPNSPRAE   177
FLR            FACSKKMTGKSIQPENLEYRIMLSVH-GSQHSGMIVNDTGHETDENRAKVEITPNSPRAE   177
R103451        FACSKKMTGKSIQPENLEYRIMLSVH-GSQHSGMIVNDTGHETDENRAKVEITPNSPRAE   177
PRVABC59       FACSKKMTGKSIQPENLEYRIMLSVH-GSQHSGMIVNDTGHETDENRAKVEITPNSPRAE   177
PLCal_ZV       FACSKKMTGKSIQPENLEYRIMLSVH-GSQHSGMIVNDTGHETDENRAKVEITPNSPRAE   177
IbH 30656      FTCSKKMTGKSIQPENLEYRIMLSVH-GSQHSGMIV------NDENRAKVEVTPNSPRAE   171
MEX 2-81       FACSKKMTGKSIQPENLEYRIMLSVH-GSQHSGMIVNDTGHETDENRAKVEITPNSPRAE   177
MR 766         FTCSKKMTGKSIQPENLEYRIMLSVH-GSQHSGM----IGYETDENRAKVEVTPNSPRAE   173
DENV-1-V1792   FKCVTKLEGKIVQYENLKYSVIVTVHTGDQHQ------VGNESTEHGTTATITPQAPTTE   174
DENV-2-V594    FTCKKNMEGKVVLPENLEYTIVITPHSGEEHA------VGNDTGKHGKEIKITPQSSITE   172
DENV-3-V1043   FQCLEPIEGKVVQYENLKYTVIITVHTGDQHQ------VGNET--QGVTAEITPQASTTE   170
```

```
R116265        ATLGGFGSLGLDCEPRTGLDFSDLYYLTMNNKHWLVHKEWFHDIPLPWHAGADTGTPHWN   237
PAN2016        ATLGGFGSLGLDCEPRTGLDFSDLYYLTMNNKHWLVHKEWFHDIPLPWHAGADTGTPHWN   237
PAN2015        ATLGGFGSLGLDCEPRTGLDFSDLYYLTMNNKHWLVHKEWFHDIPLPWHAGADTGTPHWN   237
FLR            ATLGGFGSLGLDCEPRTGLDFSDLYYLTMNNKHWLVHKEWFHDIPLPWHAGADTGTPHWN   237
R103451        ATLGGFGSLGLDCEPRTGLDFSDLYYLTMNNKHWLVHKEWFHDIPLPWHAGADTGTPHWN   237
PRVABC59       ATLGGFGSLGLDCEPRTGLDFSDLYYLTMNNKHWLVHKEWFHDIPLPWHAGADTGTPHWN   237
PLCal_ZV       ATLGGFGSLGLDCEPRTGLDFSDLYYLTMNNKHWLVHKEWFHDIPLPWHAGADTGTPHWN   237
IbH 30656      ATLGGFGSLGLDCEPRTGLDFSDLYYLTMNNKHWLVHKEWFHDIPLPWHSGADTETPHWN   231
MEX 2-81       ATLGGFGSLGLDCEPRTGLDFSDLYYLTMNNKHWLVHKEWFHDIPLPWHAGADTGTPHWN   237
MR 766         ATLGGFGSLGLDCEPRTGLDFSDLYYLTMNNKHWLVHKEWFHDIPLPWHAGADTGTPHWN   233
DENV-1-V1792   IQLTDYGALTLDCSPRTGLDFNEMVLLTMKEKSWLVHKQWFLDLPLPWTSGASTSQETWN   234
DENV-2-V594    AELTGYGTVTMECSPRTGLDFNEMVLLQMEDKAWLVHRQWFLDLPLPWLPGADTQGSNWI   232
DENV-3-V1043   AILPEYGTLGLECSPRTGLDFNEMILLTMKNKAWMVHRQWFFDLPLPWTSGATTETPTWN   230
```

```
R116265        NKEALVEFKDAHAKRQTVVVLGSQEGAVHTALAGALEAEMDGAKGRLSSGHLKCRLKMDK   297
PAN2016        NKEALVEFKDAHAKRQTVVVLGSQEGAVHTALAGALEAEMDGAKGRLSSGHLKCRLKMDK   297
PAN2015        NKEALVEFKDAHAKRQTVVVLGSQEGAVHTALAGALEAEMDGAKGRLSSGHLKCRLKMDK   297
FLR            NKEALVEFKDAHAKRQTVVVLGSQEGAVHTALAGALEAEMDGAKGRLSSGHLKCRLKMDK   297
R103451        NKEALVEFKDAHAKRQTVVVLGSQEGAVHTALAGALEAEMDGAKGRLSSGHLKCRLKMDK   297
PRVABC59       NKEALVEFKDAHAKRQTVVVLGSQEGAVHTALAGALEAEMDGAKGRLSSGHLKCRLKMDK   297
PLCal_ZV       NKEALVEFKDAHAKRQTVVVLGSQEGAVHTALAGALEAEMDGAKGRLSSGHLKCRLKMDK   297
IbH 30656      NKEALVEFKDAHAKRQTVVVLGSQEGAVHTALAGALEAEMDGAKGRLSSGHLKCRLKMDK   291
MEX 2-81       NKEALVEFKDAHAKRQTVVVLGSQEGAVHTALAGALEAEMDGAKGRLSSGHLKCRLKMDK   297
MR 766         NKEALVEFKDAHAKRQTVVVLGSQEGAVHTALAGALEAEMDGAKGKLFSGHLKCRLKMDK   293
DENV-1-V1792   RQDLLVTFKTAHAKKQEVVVLGSQEGAMHTALTGATEIQTSGTT-TIFAGHLKCRLKMDK   293
DENV-2-V594    QKETLVTFKNPHAKKQDVVVLGSQEGAMHTALTGATEIQMSSGN-LLFTGHLKCRLRMDK   291
DENV-3-V1043   RKELLVTFKNAHAKKQEVVVLGSQEGAMHTALTGATEIQNSGGT-SIFAGHLKCRLKMDK   289

R116265        LRLKGVSYSLCTAAFTFTKIPAETLHGTVTVEVQYAGTDGPCKVPAQMAVDMQTLTPVGR   357
PAN2016        LRLKGVSYSLCTAAFTFTKIPAETLHGTVTVEVQYAGTDGPCKVPAQMAVDMQTLTPVGR   357
PAN2015        LRLKGVSYSLCTAAFTFTKIPAETLHGTVTVEVQYAGTDGPCKVPAQMAVDMQTLTPVGR   357
FLR            LRLKGVSYSLCTAAFTFTKIPAETLHGTVTVEVQYAGTDGPCKVPAQMAVDMQTLTPVGR   357
R103451        LRLKGVSYSLCTAAFTFTKIPAETLHGTVTVEVQYAGTDGPCKVPAQMAVDMQTLTPVGR   357
PRVABC59       LRLKGVSYSLCTAAFTFTKIPAETLHGTVTVELQYAGTDGPCKVPAQMAVDMQTLTPVGR   357
PLCal_ZV       LRLKGVSYSLCTAAFTFTKIPAETLHGTVTVEVQYAGTDGPCKVPAQMAVDMQTLTPVGR   357
IbH 30656      LRLKGVSYSLCTAAFTFTKVPAETLHGTVTVEVQYAGRDGPCKVPAQMAVDMQTLTPVGR   351
MEX 2-81       LRLKGVSYSLCTAAFTFTKIPAETLHGTVTVEVQYAGTDGPCKVPAQMAVDMQTLTPVGR   357
MR 766         LRLKGVSYSLCTAAFTFTKVPAETLHGTVTVEVQYAGTDGPCKIPVQMAVDMQTLTPVGR   353
DENV-1-V1792   LTLKGMSYVMCTGSFKLEKEVAETQHGTVLVQIKYEGTDAPCKIPFSTQ-DEKGVTQNGR   352
DENV-2-V594    LQLKGMSYSMCTGKFKIVKEIAETQHGTIVIRVQYEGDGSPCKIPFEIM-DLEKRHVLGR   350
DENV-3-V1043   LELKGMSYAMCTNTFVLKKEVSETQHGTILIKVEYKGEDAPCKIPFSTE-DGQGKAHNGR   348

R116265        LITANPVITESTENSKMMLELDPPFGDSYIVIGVGEKKITHHWHRSGSTIGKAFEATVRG   417
PAN2016        LITANPVITESTENSKMMLELDPPFGDSYIVIGVGEKKITHHWHRSGSTIGKAFEATVRG   417
PAN2015        LITANPVITESTENSKMMLELDPPFGDSYIVIGVGEKKITHHWHRSGSTIGKAFEATVRG   417
FLR            LITANPVITESTENSKMMLELDPPFGDSYIVIGVGEKKITHHWHRSGSTIGKAFEATVRG   417
R103451        LITANPVITESTENSKMMLELDPPFGDSYIVIGVGEKKITHHWHRSGSTIGKAFEATVRG   417
PRVABC59       LITANPVITESTENSKMMLELDPPFGDSYIVIGVGEKKITHHWHRSGSTIGKAFEATVRG   417
PLCal_ZV       LITANPVITESTENSKMMLELDPPFGDSYIVIGVGEKKITHHWHRSGSTIGKAFEATVRG   417
IbH 30656      LITANPVITESTENSKMMLELDPPFGDSYIVIGVGDKKITHHWHRSGSIIGKAFEATVRG   411
MEX 2-81       LITANPVITESTENSKMMLELDPPFGDSYIVIGVGEKKITHHWHRSGSTIGKAFEATVRG   417
MR 766         LITANPVITESTENSKMMLELDPPFGDSYIVIGVGDKKITHHWHRSGSTIGKAFEATVRG   413
DENV-1-V1792   LITANPIVTD--KEKPVNIEAEPPFGESYIVIGAGEKALKLSWFKKGSSIGKMFEATARG   410
DENV-2-V594    LITVNPIVTE--KDSPVNIEAEPPFGDSYIIIGVEPGQLKLNWFKKGSSIGQMFETTMRG   408
DENV-3-V1043   LITANPVVTK--KEEPVNIEAEPPFGESNIVIGIGDNALKINWYKKGSSIGKMFEATARG   406

R116265        AKRMAVLGDTAWDFGSVGGALNSLGKGIHQIFGAAFKSLFGGMSWFSQILIGTLLMWLGL   477
PAN2016        AKRMAVLGDTAWDFGSVGGALNSLGKGIHQIFGAAFKSLFGGMSWFSQILIGTLLMWLGL   477
PAN2015        AKRMAVLGDTAWDFGSVGGALNSLGKGIHQIFGAAFKSLFGGMSWFSQILIGTLLMWLGL   477
FLR            AKRMAVLGDTAWDFGSVGGALNSLGKGIHQIFGAAFKSLFGGMSWFSQILIGTLLMWLGL   477
R103451        AKRMAVLGDTAWDFGSVGGALNSLGKGIHQIFGAAFKSLFGGMSWFSQILIGTLLMWLGL   477
PRVABC59       AKRMAVLGDTAWDFGSVGGALNSLGKGIHQIFGAAFKSLFGGMSWFSQILIGTLLMWLGL   477
PLCal_ZV       AKRMAVLGDTAWDFGSVGGALNSLGKGIHQIFGAAFKSLFGGMSWFSQILIGTLLMWLGL   477
IbH 30656      AKRMAVLGDTAWDFGSVGGVFNSLGKGIHQIFGAAFKSLFGGMSWFSQILIGTLLVWLGL   471
MEX 2-81       AKRMAVLGDTAWDFGSVGGALNSLGKGIHQIFGAAFKSLFGGMSWFSQILIGTLLMWLGL   477
MR 766         AKRMAVLGDTAWDFGSVGGVFNSLGKGIHQIFGAAFKSLFGGMSWFSQILIGTLLVWLGL   473
DENV-1-V1792   ARRMAILGDTAWDFGSIGGVFTSVGKLVHQIFGTAYGVLFSGVSWTMKIGIGVLLTWLGL   470
DENV-2-V594    AKRMAILGDTAWDFGSLGGVFTSIGKALHQVFGAIYGAAFSGVSWTMKILIGVIITWIGM   468
DENV-3-V1043   ARRMAILGDTAWDFGSVGGVLNSLGKMVHQIFGSAYTALFSGVSWVMKIGIGVLLTWIGL   466

R116265        NTKNGSISLMCLALGGVLIFLSTAVSA   504
PAN2016        NTKNGSISLMCLALGGVLIFLSTAVSA   504
PAN2015        NTKNGSISLMCLALGGVLIFLSTAVSA   504
FLR            NTKNGSISLMCLALGGVLIFLSTAVSA   504
R103451        NTKNGSISLMCLALGGVLIFLSTAVSA   504
PRVABC59       NTKNGSISLMCLALGGVLIFLSTAVSA   504
PLCal_ZV       NTKNGSISLMCLALGGVLIFLSTAVSA   504
IbH 30656      NTKNGSISLTCLALGGVMIFLSTAVSA   498
MEX 2-81       NTKNGSISLMCLALGGVLIFLSTAVSA   504
MR 766         NTKNGSISLTCLALGGVMIFLSTAVSA   500
DENV-1-V1792   NSRSTSLSMTCIAVGLVTLYLGVMVQA   497
DENV-2-V594    NSRSTSLSVSLVLVGVVTLYLGVMVQA   495
DENV-3-V1043   NSKNTSMSFSCIAIGVITLYLGAVVQA   493
```

Figure S3. Multiple sequence alignment of amino acid (aa) sequences of E protein of 10 ZIKV strains and DENV-1-3 human strains used in this study. Schematic maps of ZIKV polyprotein and ZIKV E protein are listed on top of the alignment. Amino acids variable positions are highlighted. The sequence of DENV-4-PR 06-65-740 is not available, so its sequence alignment is not included. The alignment was performed using Clustal V method of MegAlign (v7.1) within the DNASTAR package. C: capsid; prM: precursor of membrane; E: envelope; NS1, NS2A, NS2B, NS3, NS4A, NS4B, and NS5: nonstructural proteins. DI-DIII: domain I-III of E protein; FL: fusion loop; S: stalk region; TM: transmembrane domain.
